# Cpt1c Downregulation Causes Plasma Membrane Remodelling and Anthracycline Resistance in Breast Cancer

**DOI:** 10.3390/ijms24020946

**Published:** 2023-01-04

**Authors:** Helena Muley, Karmele Valencia, Josefina Casas, Bea Moreno, Luis Botella, Fernando Lecanda, Rut Fadó, Núria Casals

**Affiliations:** 1 Basic Sciences Department, Faculty of Medicine and Health Sciences, Universitat Internacional de Catalunya, 08195 Sant Cugat del Vallès, Spain; 2 Program in Solid Tumors, Center for Applied Medical Research (CIMA), University of Navarra, 31008 Pamplona, Spain; 3 Centro de Investigación Biomédica en Red de Cáncer (CIBERONC), 28029 Madrid, Spain; 4 Department of Biochemistry and Genetics, School of Sciences, University of Navarra, 31008 Pamplona, Spain; 5 Research Unit on Bioactive Molecules (RUBAM), Department of Biological Chemistry, Institute for Advanced Chemistry of Catalonia (IQAC-CSIC), Spanish National Research Council (CSIC), 08034 Barcelona, Spain; 6 Centro de Investigación Biomédica en Red de Enfermedades Hepáticas y Digestivas (CIBEREHD), Instituto de Salud Carlos III, 28029 Madrid, Spain; 7 Molecular Therapeutics Program, Center for Applied Medical Research (CIMA), University of Navarra, 31008 Pamplona, Spain; 8 Department of Pathology, Anatomy and Physiology, University of Navarra, 31008 Pamplona, Spain; 9 Institut de Neurociències, Universitat Autònoma de Barcelona, Bellaterra, 08193 Cerdanyola del Vallès, Spain; 10 Centro de Investigación Biomédica en Red de Fisiopatología de la Obesidad y la Nutrición (CIBEROBN), Instituto de Salud Carlos III, 28029 Madrid, Spain

**Keywords:** CPT1C, chemoresistance, drug uptake, breast cancer, doxorubicin, plasma membrane, lipid remodelling, fatty acid saturation

## Abstract

Breast cancer (BC) is the most common malignancy in women worldwide. While the main systemic treatment option is anthracycline-containing chemotherapy, chemoresistance continues to be an obstacle to patient survival. Carnitine palmitoyltransferase 1C (CPT1C) has been described as a poor-prognosis marker for several tumour types, as it favours tumour growth and hinders cells from entering senescence. At the molecular level, CPT1C has been associated with lipid metabolism regulation and important lipidome changes. Since plasma membrane (PM) rigidity has been associated with reduced drug uptake, we explored whether CPT1C expression could be involved in PM remodelling and drug chemoresistance. Liquid chromatography-high resolution mass spectrometry (LC-HRMS) lipid analysis of PM-enriched fractions of MDA-MB-231 BC cells showed that CPT1C silencing increased PM phospholipid saturation, suggesting a rise in PM rigidity. Moreover, CPT1C silencing increased cell survival against doxorubicin (DOX) treatment in different BC cells due to reduced drug uptake. These findings, further complemented by ROC plotter analysis correlating lower CPT1C expression with a lower pathological complete response to anthracyclines in patients with more aggressive types of BC, suggest CPT1C as a novel predictive biomarker for BC chemotherapy.

## 1. Introduction

Breast cancer (BC) is the most common malignancy and the second leading cause of cancer-related deaths in women worldwide [1,2]. Although BC-related survival rates have dramatically improved over the past decades, once BC becomes metastatic, it is virtually incurable [3]. The presence or absence of certain molecular markers, namely, estrogen receptor (ER), progesterone receptor (PR), and human epidermal growth factor 2 (HER2), is used to classify BC as follows: hormone receptor (HR) positive/HER2 negative (HR+/HER2-) BC, occurring in around 70% of patients; HER2 positive (HR+/−/HER2+) BC, accounting for 15–20% of patients; and triple-negative (HR−/HER2−) BC (TNBC), occurring in 15% of patients, a frequently recurring BC, and the most aggressive and lethal BC subtype in women. 

The receptor signature explains the distinct treatments used for different BC subtypes. For ER+ and PR+ BC patients, endocrine agents are the first-line systemic therapy, in some cases accompanied by chemotherapy. Patients with overexpressed HER2 benefit from targeted therapy, including anti-HER2 antibodies, usually combined with chemotherapy. For TNBC, the only systemic therapy with demonstrated efficacy is chemotherapy [4]. Of the BC chemotherapy drugs, anthracyclines are considered a gold standard, with strong evidence of their positive impact on survival [5]. However, patients treated with anthracyclines commonly develop resistance, and once resistance develops, few treatment options remain [6]. The mechanisms that contribute to the cytotoxic effect of anthracyclines are DNA intercalation, inhibition of topoisomerase II, and the generation of free radicals leading to apoptotic cell death [7].

Cancer cells, in aggressive conditions, can adapt their metabolism to support rapid proliferation, continuous growth, and survival, reprogramming their metabolism to obtain the energy and biosynthetic intermediates that are necessary to preserve their integrity [8]. In recent years, lipids have emerged as central players in cancer cell metabolic reprogramming. Changes in the composition and fate of lipids support key oncogenic functions such as cellular energetics, oncogenic signalling, and immune system response attenuation and resistance to stress and chemotherapy [9]. Recent findings have suggested that proteins regulating fatty acid (FA) transport from cytosol to mitochondria, such as carnitine palmitoyltransferase 1 (CPT1) enzymes, may play a role in cancer cell reprogramming [10,11,12,13,14,15,16,17]. 

The CPT1 protein family includes CPT1A, CPT1B, and CPT1C. Although CPT1C exhibits high sequence similarity to CPT1A and CPT1B, it is, in fact, a pseudoenzyme with a different molecular function from the canonical CPT1s [18]. CPT1C, which is only expressed in neurons and stem cells in healthy individuals [19], is located in the endoplasmic reticulum [20] and has very low catalytic activity [21]. In 2011, Zaugg et al. [10] identified CPT1C as a frequently expressed gene in human cancers, including BC and lung cancer. Subsequent CPT1C mRNA expression studies in a wide array of tumour types showed that CPT1C is also highly expressed in brain tumours and several sarcomas [11]. CPT1C overexpression is associated with higher in vitro cell survival and in vivo tumour growth, especially in conditions of metabolic stress (hypoxia or glucose deprivation) [10,12,13,22,23,24,25] and also with poorer overall survival [24,26]. CPT1C has also been defined as a novel regulator of cancer cell senescence. Silencing of CPT1C in different tumour cell lines triggers cellular senescence and mitochondrial dysfunction and inhibits tumorigenesis in vivo. In contrast, CPT1C gain-of-function has been shown to reverse cell senescence and to enhance mitochondrial function and FA oxidation (FAO) [12,14,17,22,23]. Those processes have been associated with significant lipidome changes and with lipotoxicity, demonstrating that CPT1C silencing dramatically increases the total fat content of tumour cells, and particularly of certain saturated FAs [22]. 

Given that lipid saturation changes in the plasma membrane (PM) are associated with drug chemoresistance in several tumours [27,28,29], we explored whether CPT1C expression could be involved in drug sensitivity loss in BC.

We demonstrate that CPT1C silencing drives PM lipid remodelling by increasing phospholipid saturation and chain length and promotes drug impermeability and chemoresistance in BC cells. We also find that CPT1C downregulation is both a poor-prognosis marker and a poor predictive marker for anthracycline treatment of patients with HER2+ BC and TNBC, i.e., the patients who usually receive chemotherapy.

## 2. Results

### 2.1. CPT1C Silencing Increases Phospholipid Saturation and Chain Length in the PM of MDA-MB-231 Cells

**Figure 1:**
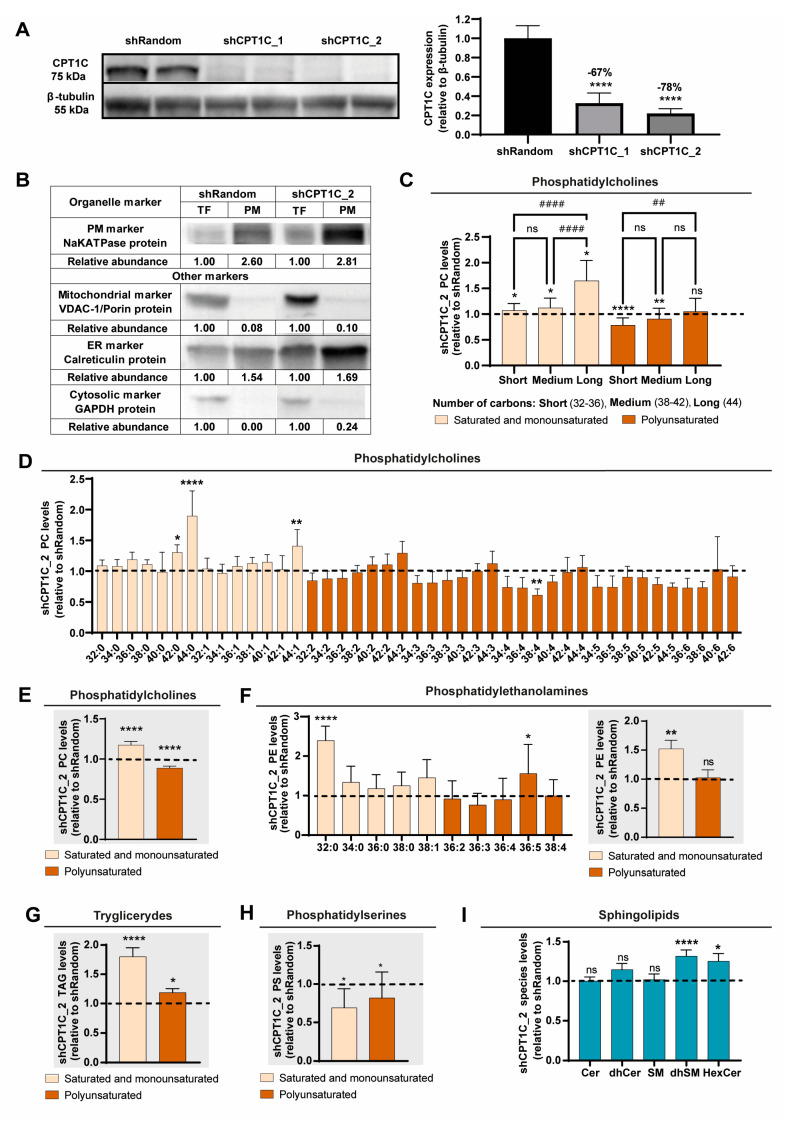
Relative abundance of lipid molecular species in a plasma membrane (PM) fraction of CPT1C-silenced MDA-MB-231 cells measured by liquid chromatography-high resolution mass spectrometry (LC-HRMS). MDA-MB-231 cells were infected with shRandom or shCPT1C_2-carrying lentivirus. (**A**) CPT1C silencing was confirmed by western blot. Tubulin was used as a loading control. Results are shown as mean ± SD from an experiment performed with 4 replicates (1-way ANOVA followed by Dunn’s multiple comparison test; **** *p* < 0.0001). (**B**) Western blot showing PM enrichment in the isolated fractions (PM) compared with total fraction (TF). Na^+^/K^+^-ATPase, VDAC-1/Porin, calreticulin, and GAPDH were selected as PM, mitochondria, endoplasmic reticulum (ER) and cytosolic protein markers, respectively. PM fractions show clear enrichment in the PM marker, an almost complete removal of mitochondrial and cytosolic markers, and a slight increase in the ER marker. (**C**–**I**) Relative abundance of lipid species in PM-enriched fractions of CPT1C-silenced MDA-MB-231 cells relative to control cells (value = 1, dashed line). Values are grouped by fatty acid (FA) chain length and saturation. In (**D**,**F**), relative abundance of specific FAs is shown. (**C**,**D**,**F**) (left). Results are shown as mean ± SD for 3 independent experiments (2-way ANOVA followed by Dunnett’s multiple comparison test; * *p* < 0.05, ** *p* < 0.01, **** *p* < 0.0001, ## *p* < 0.01, #### *p* < 0.0001), ns = non significative. (**E**,**F**) (right), (**G**,**H**,**I**) Results are shown as mean ± SEM (±SD in H) for 3 independent experiments (Wilcoxon signed-rank test; * *p* < 0.05, **** *p* < 0.0001).

To explore whether CPT1C-mediated lipidome changes, as described in the literature [22], could affect the composition of PM in BC cells, we selected the MDA-MB-231 cell line as a model of TNBC [30], and inhibited CPT1C using two short-hairpin (sh) RNAs: shCPT1C_1 and shCPT1C_2 (Figure 1A). We then analysed the lipidome of the PM-enriched cellular fraction in both control and CPT1C-inhibited MDA-MB-231 cells. PM-enriched cellular fraction was obtained by sub-cellular fractionation and was confirmed by western blot (Figure 1B). 

We found that CPT1C silencing slightly modified the relative abundance of the different lipid species in PM-enriched fractions of MDA-MB-231 cells (Figure S1A). However, more remarkably, we found a notable difference in phospholipid saturation and chain length, especially for phosphatidylcholine (PC) and phosphatidylethanolamine (PE), the most abundant phospholipids in mammalian cell membranes. The PC species contained longer and more saturated FAs in the CPT1C-silenced cells compared to the control cells. Figure 1C,D depict the levels of PC species found in CPT1C-silenced cells compared to control cells (value = 1). In general, PC species with polyunsaturated FAs were diminished, while PC species with saturated and monounsaturated FAs were increased (Figure 1E); similar results were obtained for the PE species (Figure 1F). For CPT1C-silenced MDA-MB-231 cells, we consistently found enhanced saturation of the FAs that form triglycerides associated with the PM (Figure 1G). 

We also calculated the PC/PE ratio, a marker of PM fluidity, for the MDA-MB-231 cells, consistently finding that CPT1C silencing significantly decreased this ratio (Figure S1B). Similar to their corresponding precursors, saturated and monounsaturated lysoglycerophospholipids were increased in the PM-enriched fractions of shCPT1C-MDA-MB-231 cells (Figure S1C). Considering that the PC and PE species are the most abundant lipids in the PM, and that lipid saturation level and chain length are key determinants of PM fluidity and permeability, our results suggest that CPT1C silencing decreases PM fluidity in MDA-MB-231 cells.

As for phosphatidylserine (PS), under CPT1C silencing this was significantly decreased, independently of the extent of FA saturation (Figure 1H). Since PS has a net negative charge at physiological pH, the fact that it is less present in shCPT1C-MDA-MB-231 cells may be indicative of a less negatively charged PM, which, in turn, may alter the uptake of positively charged drugs, such as doxorubicin (DOX) in the cancer environment.

Even though PM cholesterol is closely related to PM rigidity, total cholesterol levels remained unchanged in our CPT1C-silenced cells (Figure S1D). Regarding sphingolipids, in CPT1C-silenced MDA-MB-231 cells compared to control cells, we found no change in ceramide (Cer), dihydroceramide (dhCer), or sphingomyelin (SM) levels, but did find increased hexosylceramide (HexCer) and dihydrosphingomyelin (dhSM) levels (Figure 1I). Both the dhSM and HexCer species are critical lipid constituents of lipid raft microdomains [31,32], and the dhCer/Cer and dhSM/SM ratios are indicative of the saturation level of the sphingosine backbone. CPT1C silencing increased the dhSM/SM ratio (by 50%), while the dhCer/Cer ratio and the relative SM and Cer proportions remained unchanged (Figure S1E). Considering that dhSM forms, via effective hydrogen bond formation, more condensed and ordered membrane domains than SM, and that SM is much more abundant than Cer in the PM [33], an increased dhSM/SM ratio may contribute to increased PM rigidity in CPT1C-silenced MDA-MB-231 cells. 

### 2.2. CPT1C Silencing Impairs DOX Uptake in MDA-MB-231 Cells

The intense remodelling of PM lipid composition caused by CPT1C silencing in MDA-MB-231 cells points to a possible decrease in PM permeability to external molecules entering the cells through passive diffusion. The entry of chemotherapeutic agents such as DOX, one of the most efficacious treatments for both early- and late-stage BC, is highly dependent on PM lipid composition [34]. For this reason, we explored whether CPT1C silencing impaired DOX uptake in MDA-MB-231 cells.

**Figure 2:**
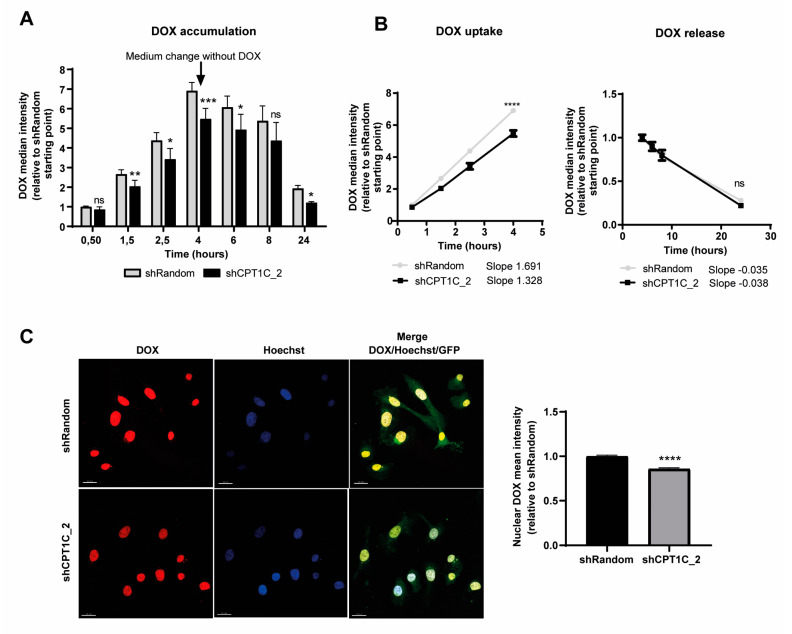
CPT1C silencing decreases doxorubicin (DOX) cellular and nuclear accumulation by decreasing uptake. MDA-MB-231 cells were infected with shRandom or shCPT1C_2-carrying lentivirus. (**A**) Quantification of intracellular DOX at the indicated times as detected by flow cytometry. Cells were treated with 1 µg/mL DOX for 4 h, after which the medium was changed to remove DOX and study drug release until 24 h. Results are shown as median ± SD for 2 independent experiments performed with 4 biological replicates (*n* = 8; 2-way ANOVA followed by Bonferroni’s multiple comparison test; * *p* < 0.05, ** *p* < 0.01, *** *p* < 0.001). ns = non significative. (**B**) DOX accumulation kinetics (uptake and normalized release) from both flow cytometry experiments. Slopes for both were compared (*n* = 8, ANCOVA for slope comparison; uptake **** *p* < 0.0001 and release *p* = 0.4366). (**C**) (left). Intracellular localization of DOX visualized by confocal microscopy 2 h 30 min after DOX exposure. Nuclei were labelled with Hoechst (scale bars, 20 μm). (**C**) (right). Quantification of fluorescence intensity (DOX) in the nuclei. Results are shown as mean ± SEM for 2 independent experiments performed with 4 biological replicates (*n* = 245–289 cells; Mann–Whitney test; **** *p* < 0.0001).

Taking advantage of the inherent fluorescent properties of DOX and using flow cytometry with the pulse-chase method, we directly analysed intracellular DOX accumulation in sh-random and CPT1C-silenced MDA-MB-231 cells. We incubated cells with DOX for 4 h, then changed the medium to keep the cells in a drug-free medium for a further 20 h. As expected, intracellular DOX accumulation increased in the first 4 h, and then decreased in a time-dependent manner after the medium replacement. However, CPT1C silencing with both sh-CPT1C sequences (shCPT1C_1 and shCPT1C_2) significantly reduced DOX accumulation in MDA-MB-231 cells compared to control cells (Figure 2A and Supplementary Figure S2).

Pharmacokinetic analysis of drug uptake (from 0 to 4 h) and drug release (from 4 to 24 h) revealed significant differences in drug-uptake but not drug-release slopes, indicating that the reduced DOX accumulation in CPT1C-silenced MDA-MB-231 cells was caused by a drug-uptake impairment (Figure 2B and Supplementary Figure S2A). Confocal microscopy analysis of DOX nuclear accumulation in MDA-MB-231 cells confirmed that less drug reached its tagged compartment under CPT1C silencing (Figure 2C and Supplementary Figure S2B). Those results suggest that CPT1C depletion impairs the uptake of DOX and other chemotherapy agents in TNBC cells.

### 2.3. CPT1C Silencing Promotes Resistance to DOX in BC Cells

**Figure 3:**
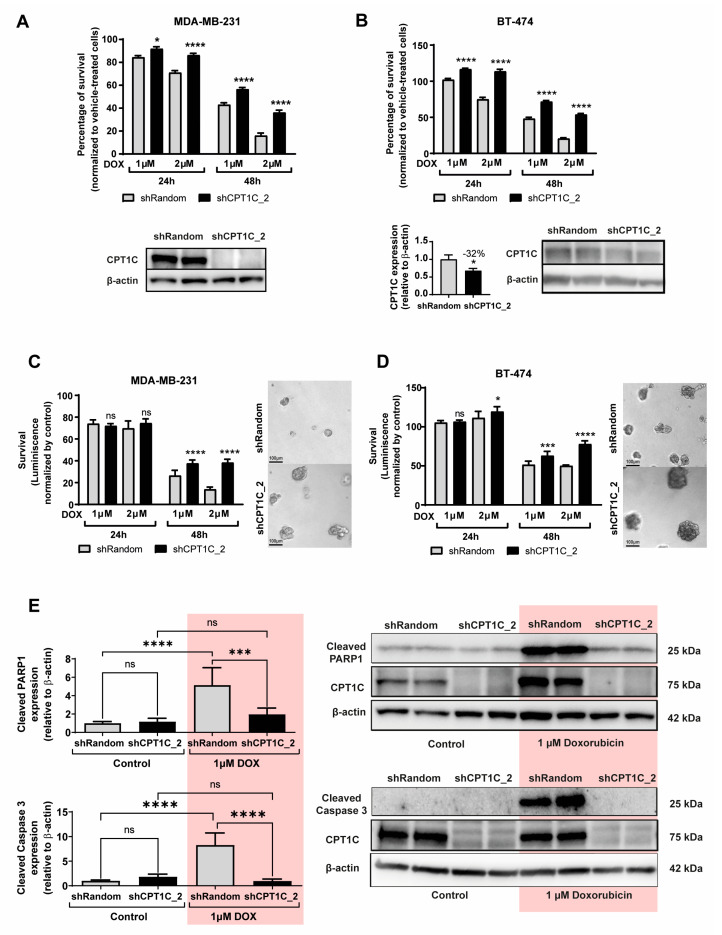
CPT1C silencing increases DOX resistance in breast cancer (BC) cells. MDA-MB-231 (**A**,**C**) and BT-474 (**B**,**D**) cells were infected with shRandom or shCPT1C-carrying lentivirus. (**A**,**B**) (above). The MTT assay evaluation of BC cell line chemosensitivity to DOX after 24 or 48 h of treatment at the indicated doses. Results are shown as mean ± SEM for 3 independent experiments performed with 4 biological replicates (*n* = 12; 2-way ANOVA followed by Bonferroni’s multiple comparison test; * *p* < 0.05, **** *p* < 0.0001). (**A**,**B**) (below). CPT1C silencing confirmed by western blot. β-actin was used as a loading control. Quantification of CPT1C silencing in BT-474 cell line is included (*n* = 4; Student *t* test; * *p* < 0.05). (**C**,**D**) Cell viability of MDA-MB-231 and BT-474 mammospheres was determined by CellTiter-Glo^®^ luminescent cell viability assay. A representative image of each cell line is shown. Images represent mammospheres 24 h after 2 μM DOX treatment. Results are shown as mean ± SD for 2 independent experiments (*n* = 9 and *n* = 6, respectively; 2-way ANOVA followed by Bonferroni’s multiple comparison test; * *p* < 0.05, *** *p* < 0.001, **** *p* < 0.0001). ns = non significative. (**E**) Western blot analysis of apoptosis-associated proteins (cleaved PARP1 and cleaved caspase 3). Cell lysates were prepared after 24 h of DOX treatment. CPT1C silencing was confirmed and β-actin was used as a loading control. Representative results for 2 independent experiments are shown as mean ± SD (*n* = 4 per condition; 1-way ANOVA followed by Dunn’s multiple comparison test; *** *p* < 0.001, **** *p* < 0.0001).

We also analysed whether CPT1C silencing caused chemoresistance in BC cells. Interestingly, cell viability assays showed that DOX toxicity was clearly reduced under CPT1C silencing in MDA-MB-231 cells for both the sh-CPT1C sequences used (sh-CPT1C_2 in Figure 3A and sh-CPT1C_1 in Figure S3A).

Similar results were obtained for luminal B (ER+, PR+, HER2+) BT-474 (Figure 3B), luminal A (ER+, PR+, HER2−) MCF7 (Supplementary Figure S3B), and TNBC Hs578T and CAL-51 (Supplementary Figure S3C) cells. Interestingly, CPT1C silencing also caused resistance to paclitaxel (taxane) in MDA-MB-231 cells (Supplementary Figure S3D), but not in BT-474 or MCF7 cells (Supplementary Figure S3E). In order to generate a more physiological in vitro model, we cultured 3D mammospheres with MDA-MB-231 and BT-474 cells, and, using the same time-points and doses of DOX as used for the 2D cultures, ascertained DOX resistance using a 3D cell viability assay (CellTiter-Glo^®^, Promega, Madison, WI, USA). Similar to results for the 2D cultures, CPT1C silencing increased DOX resistance in mammosphere cultures of both cell lines (Figure 3C,D). To confirm this chemoresistance effect, using western blot we measured apoptosis activation (the cleavage of PARP1 and caspase-3), which is one of the DOX action mechanisms. As expected, CPT1C silencing with both shCPT1C sequences was capable of fully preventing apoptosis activation at 24 h of DOX treatment in MDA-MB-231 cells (Figure 3E and Supplementary Figure S3F). 

### 2.4. CPT1C Silencing Impairs Liposomal-DOX Uptake and Sensitivity in MDA-MB-231 Cells

**Figure 4:**
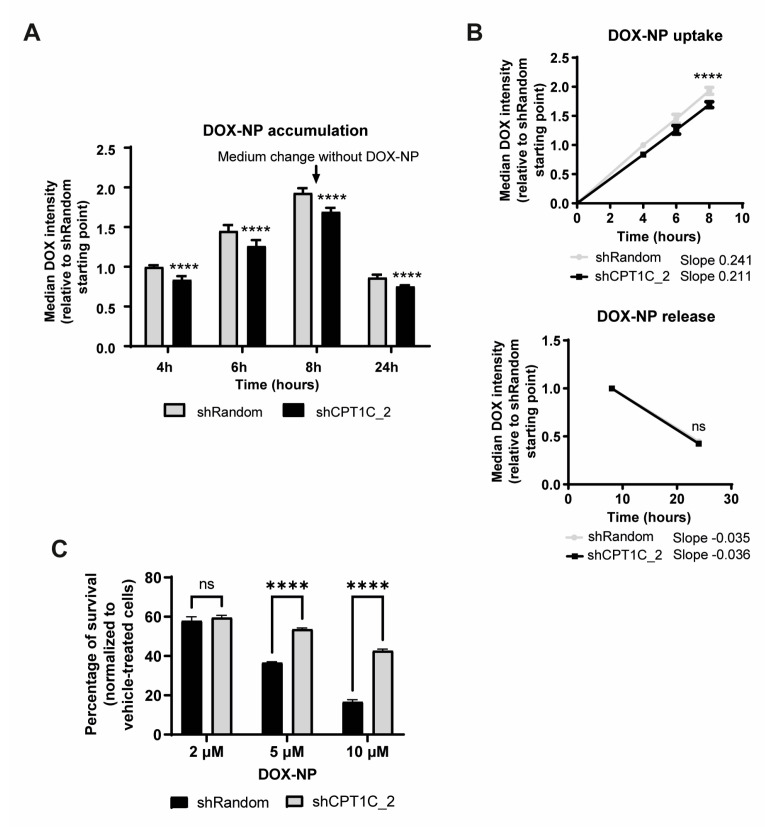
CPT1C silencing impairs liposomal-DOX uptake and sensitivity in MDA-MB-231 cells. MDA-MB-231 cells were infected with shRandom or shCPT1C-carrying lentivirus. (**A**) Quantification of intracellular liposomal-DOX at the indicated times as detected by flow cytometry. Cells were treated with 1 µg/mL liposomal-DOX for 8 h, after which the medium was changed to remove liposomal-DOX and study drug release until 24 h. Results are shown as median ± SD for 2 independent experiments performed with 4 biological replicates (*n* = 8, 2-way ANOVA followed by Bonferroni’s multiple comparison test; **** *p* < 0.0001). (**B**) Liposomal-DOX accumulation kinetics (uptake and normalized release) from both flow cytometry experiments. Slopes of both conditions were compared (*n* = 8, analysis of covariance (ANCOVA) for slope comparison; uptake **** *p* < 0.0001 and release *p* = 0.2063). ns = non significative. (**C**) MTT assay evaluation of MDA-MB-231 cell chemosensitivity to liposomal-DOX after 48 h of treatment at the indicated doses. Results represent mean ± SEM for 3 independent experiments (*n* = 12, 2-way ANOVA followed by Bonferroni’s multiple comparison test; **** *p* < 0.0001). ns = non significative.

It has been reported that the rigid nature of a resistant cellular membrane not only affects free drug uptake, but also the endocytic function by which drug nanoparticles enter cells. To avoid the frequent cardiologic side effects of DOX, liposome-encapsulated DOX (liposomal-DOX) is currently being used in clinical practice as monotherapy for metastatic BC. To study whether CPT1C silencing could also impair the uptake and sensitivity of liposomal-DOX in MDA-MB-231 cells, we incubated MDA-MB-231 cells with liposomal-DOX for 8 h, then changed the medium and incubated the cells in a drug-free medium for a further 16 h. A drug accumulation flow cytometry graph revealed that CPT1C silencing significantly reduced DOX accumulation in MDA-MB-231 cells (Figure 4A), due to impaired drug uptake, but not drug release (Figure 4B). We then measured drug toxicity in MDA-MB-231 cells by treating them with 2 μM, 5 μM, and 10 μM of liposomal-DOX for 48 h. Cell viability assays showed that CPT1C silencing clearly decreased liposomal-DOX toxicity in a dose-dependent manner (Figure 4C). Overall, those results suggest that PM remodelling caused by CPT1C downregulation alters drug entry by both passive diffusion and endocytosis processes.

### 2.5. CPT1C as a Predictive Biomarker of Anthracycline Response Is of Prognostic Value for Patients with the TNBC and HER2+ BC Subtypes 

**Figure 5:**
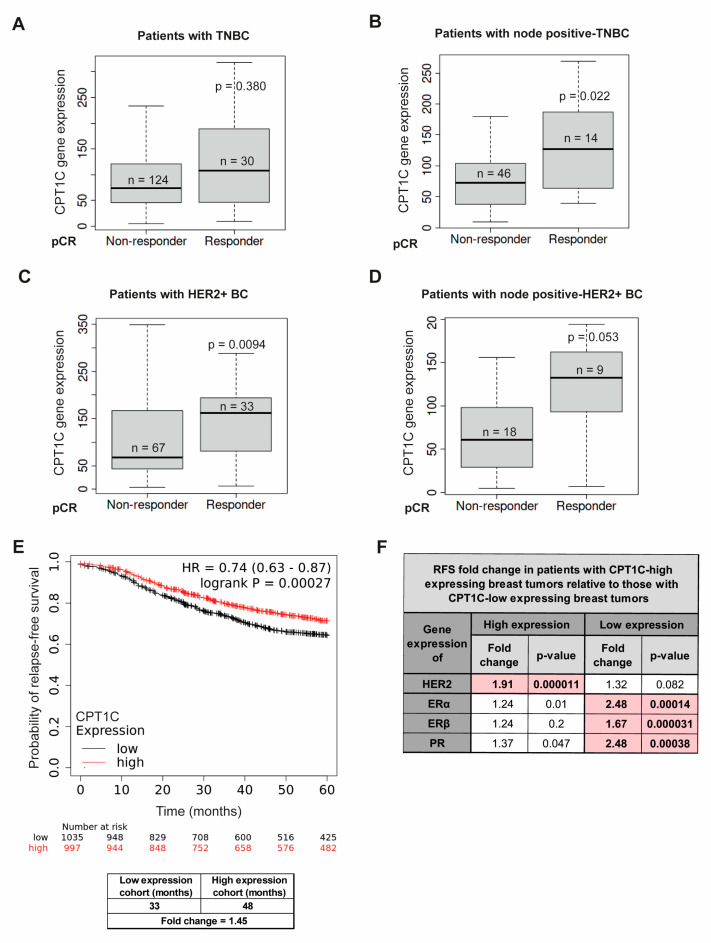
Low CPT1C expression is associated with poorer pathological complete response (pCR) to anthracycline treatment and poor relapse-free survival (RFS). (**A**–**D**) The ROC plotter web application (www.rocplot.org; 22 February 2022) was used to link CPT1C expression with therapy response (based on pCR) using the transcriptome-level data of patients with BC, filtered for anthracycline-treated patients with TNBC (**A**), node positive-TNBC (**B**), HER2+ BC (**C**), and node positive-HER2+ BC (**D**). Box-and-whisker plots represent the median, minimum, and maximum values of CPT1C expression for responding and nonresponding patients according to pCR. (**E**) RFS rate for patients with BC with low or high CPT1C gene expression as analysed using a Kaplan–Meier plotter, showing the log-rank p-value and hazard ratio (HR; 95% CI in parentheses). The corresponding Affymetrix ID for CPT1C is 227468_at. (**F**) CPT1C expression implication in RFS in patients with BC according to typical BC molecular markers. The value of RFS (months) in patients with tumours with high CPT1C expression was divided by the value of RFS (months) in patients with low CPT1C expression. This calculation was segregated depending on the expression (high or low) of different molecular markers, namely, HER2, ERα, ERβ and PR. The greatest impact of CPT1C in survival was observed in patients with high HER2 and low ERα, ERβ and PR expression.

Given the evidence of CPT1C-mediated chemoresistance in vitro, we determined whether CPT1C could be a predictive biomarker of chemotherapy failure in patients with BC. We used ROC plotter, a transcriptome-level validation tool for predictive cancer biomarkers [35], and a transcriptome database for 3104 patients with BC. In this database, patients are assigned to a responder or a non-responder cohort based on their clinical characteristics (grade, nodal status, receptor status, and molecular subtype). For the purposes of our study, patients receiving neoadjuvant chemotherapy were classified according to pathological complete response (pCR), defined as the disappearance of all invasive cancer in the breast and axillary nodes after completion of neoadjuvant therapy. pCR patients are known to have increased overall survival (OS) and relapse-free survival (RFS) [36,37]. We only analysed patients treated with anthracyclines (e.g., DOX), and filtered by the molecular subtype, first examining pCR for all patients, and then restricting the analysis to patients who were node-positive, given that anthracycline appears to be more effective in patients with greater lymph node involvement [3]. When patients with TNBC received anthracycline treatment, ROC plotter analysis showed that pCR was less likely for low CPT1C expression than for high CPT1C expression tumours, and that the difference was significant for the node-positive group (Figure 5A,B). 

Similar results were found for patients with HER2+ BC (Figure 5C,D), although the number of samples was reduced in this case. However, CPT1C expression did not show any effect in patients with HR+ tumours such as luminal A (Supplementary Figure S4). Therefore, CPT1C expression may be clinically useful as a predictive marker of anthracycline treatment specifically for patients with TNBC and HER2+ BC.

We then investigated, using a Kaplan–Meier plotter whether CPT1C expression could affect BC prognosis and patient survival. The RFS curve plots for 1764 patients with BC showed, interestingly, that high CPT1C levels were a protective factor for RFS in patients with BC (Figure 5E). We then filtered the Kaplan–Meier analysis according to expression levels of different molecular markers used to classify BC types, namely, HER2, ER alpha and beta (Erα and Erβ) and PR (Figure 5F). Results show that low CPT1C expression was related to poorer RFS rates only for tumours with high HER2 expression and tumours with low ERα, Erβ, and PR expression. Those results indicate that low CPT1C expression is a poor-prognosis marker specifically for HER2+ BC and TNBC. 

## 3. Discussion

In recent years, metabolic reprogramming has come to be considered a major hallmark in malignancy. Cancer cells adapt their metabolism to support tumour growth and development (survival in harsh conditions, invasion, metastasis, dormancy, recurrence, and chemoresistance). In the past decade, an array of publications have demonstrated how CPT1C enables cancer cells to adapt their lipid metabolism to resist stressful conditions such as hypoxia and glucose deprivation [10,13], avoid cellular senescence, and enhance tumorigenesis [12,14,17,22,23]. In this context, we explored whether CPT1C could affect lipid PM composition and be implicated in chemoresistance. We studied chemoresistance in BC cells because BC is the second cause of cancer-related deaths worldwide [1], while chemoresistance, especially in some BC types such as TNBC, is inevitable and has a poor prognosis [38]. Due to the special molecular phenotype, patients with TNBC are unable to benefit from endocrine therapy or molecular targeted therapy.

In BC treatment, while DOX is one of the most effective drugs available, chemotherapy may fail due to different chemoresistance mechanisms [39]. A mechanism observed in DOX-resistant BC cells is impaired drug uptake due to increased PM rigidity. This mechanism is especially relevant for DOX and other anthracyclines because cell entry is mainly by passive diffusion [28]. Our lipidomic results show that CPT1C depletion increases PM rigidity by remodelling its lipid composition, and so probably causes drug uptake impairment and chemoresistance. CPT1C-silenced MDA-MB-321 cells show increased saturation and length of PM phospholipids, especially in the PC species, which constitutes almost 45% of all PM lipids. Moreover, the decreased PC/PE ratio and the increase in dhSM may contribute to this rigidity. 

How can CPT1C expression trigger those changes in PM lipid composition? Since CPT1C has been demonstrated to enhance FAO [10,24,40,41], and since mitochondria primarily oxidize saturated and monounsaturated FAs, we think that the increase in those FA species under CPT1C silencing is due, at least partially, to FAO downregulation. Furthermore, since palmitate, one of the main substrates of mitochondrial FAO, is the precursor of sphingolipids, greater availability of this molecule would explain the general increase in sphingolipids observed when CPT1C is depleted. It is worth noting that CPT1C, because it does not have efficient catalytic activity, does not regulate FAO directly, but does so indirectly through an as yet unknown mechanism [42,43]. Another factor that may contribute to the increased saturation of PM phospholipids is the rise in reactive oxygen species (ROS) observed in MDA-MB-321 and other cells under CPT1C silencing [12,22]. Polyunsaturated FAs are more unstable and more easily peroxidized by ROS, leading to their faster degradation, and consequently, to an increase in the percentage of saturated phospholipids. Finally, we cannot rule out the possibility that CPT1C silencing may downregulate the activity of different lipid desaturases, as it does with other enzymes [44,45].

The decrease in PS species found in CPT1C- silenced cells may also contribute to impaired DOX uptake. DOX (pKa~7.2–8.2) is an amphiphilic molecule containing an aminosugar, which becomes positively charged in the tumour environment (acid pH) [46]. The PM negatively charged by lipid species such as PS is known to enhance DOX binding to the outer leaflet of PM prior to internalization [9].

We found, consistent with the greater rigidity of the PM in CPT1C-silenced cells, increased impermeability to DOX (both the free and liposomal forms), indicating that PM lipid remodelling not only affects drug uptake via passive diffusion but also via endocytosis. It is worth noting that we did not find changes in PM cholesterol content, which would suggest that, in our model, a change in the extent of lipid saturation was sufficient to impair drug uptake. Accordingly, DOX insensitivity and chemoresistance were observed in different BC cell lines when CPT1C was downregulated in both 2D cultures and mammospheres. 

Interestingly, clinical data agrees with the in vitro results for the BC cell lines. The ROC plotter analysis shows that CPT1C expression is lower for HER2+ BC and TNBC non-responders than for responders to anthracycline treatment. Accordingly, RFS is lower in BC patients with low CPT1C expression, especially in patients that are HER2+, ERα-, Erβ-, or PR-. Therefore, low CPT1C expression can be considered a poor predictive biomarker for anthracycline treatment in patients with HER2+ BC and TNBC. These data are of great importance, since anthracycline-based chemotherapy is currently one of the preferred therapeutic options for TNBC (the most aggressive and lethal BC type), and, along with anti-HER2 drugs, also for HER2+ BC. 

CPT1C silencing causes not only lipid remodelling, but also mitochondrial impairment and cell cycle arrest. In fact, CPT1C silencing has been proposed as a potential cancer-treatment strategy to induce senescence and halt tumour proliferation [12,16,17,47]. However, senescence is a double-edged sword that can function in opposite directions. It may serve as a pathway to tumour dormancy, defined as a latency period (possibly of years) when cancer remains in an undetectable state before emerging as an overtly proliferative disease [48,49]. Dormancy and senescence states have been clearly linked to chemoresistance [50,51]. Therefore—and even though high CPT1C expression has been demonstrated to trigger tumour growth and metastasis in different mice models [10,12,13,26], and to be associated with low OS for several human tumours [24,26]—CPT1C downregulation does not seem to be an appropriate strategy for HER2+ and TNBC since it leads to senescence and anthracyclines chemoresistance. The potential use of CPT1C inhibitors for cancer treatment would apply only to HER2- and hormone positive BC patients. Interestingly, PARP inhibitors such as olaparib, which block DNA damage response system have been recently FDA approved for HER2- BC treatment [52] and seem to potentiate DOX damaging effects [53]. The combination of CPT1C inhibitors with DOX and PARP1 inhibitors, could be advantageous for HER2- patients and should require further research. Our group is developing nanomedicine strategies to target CPT1 proteins with potential use in tumors [54]. 

In summary, our findings demonstrate that CPT1C silencing drives PM remodelling, leading, in turn, to DOX chemoresistance in BC cells, and also that low CPT1C expression is a predictive marker of poor survival for anthracycline-based neoadjuvant chemotherapy for patients with HER+ BC and TNBC. Finally, our research throws light on the key chemoresistance role played by cancer cell lipid membrane composition, and, moreover, points to the importance of a better understanding of chemoresistance mechanisms in terms of both improving long-term efficacy of anticancer therapies in human tumours and developing new targeted drugs.

## 4. Materials and Methods

### 4.1. Cell Culture

All the human BC (MDA-MB-231, MCF7, BT-474, Hs578T, CAL-51) and the HEK293T cell lines were cultured in Dulbecco’s Modified Eagle’s Medium (DMEM) (41966 Gibco, Waltham, MA, USA), supplemented with 10% foetal bovine serum (FBS-12A Capricorn Scientific, Birmingham, England), 100 U/mL penicillin, and 100 µg/mL streptomycin (15140122 Thermo Fisher, Waltham, MA, USA), and maintained at 37 °C in 5% CO2 humidified air. MDA-MB-231 cells were authenticated on the basis of viability, recovery, growth, and morphology by ATCC on 24 September 2018, and all cell lines were tested for mycoplasma contamination using the EZ-PCR™ Mycoplasma Detection Kit (20-700-20BI).

### 4.2. Plasmids and Lentiviral Infection

Cell lines showing CPT1C-shRNA silencing were generated using lentiviral infection. pLVTHM-shLVTHM1-CPT1C-IRES-GFP (shCPT1C_1), pLVTHM-shLVTHM2-CPT1C-IRE-GFP (shCPT1C_2), and pLVTHM-Random-IRES-GFP were constructed using the previously validated silencing CPT1C sequences or a random sequence [10]. The map and the sequences for these plasmids are available from Addgene (Cambridge, MA, USA) and the lentiviruses were propagated and titrated as previously described [55].

### 4.3. 3D Culture

Culture media mixed at a 1:1 ratio with growth-factor reduced Matrigel (GFR Matrigel; 356231 BD Biosciences, 9.1 mg/mL) was added to a 96-well opaque plate (136101 Thermo Fisher Scientific, Waltham, MA, USA) or clear plate, and was incubated at 37 °C for 30 min. A total of 2000 cells/well of MDA-MB-231 or 10,000 cells/well of BT-474 in 10% Matrigel medium were added on top of the coating and maintained in culture. Pictures of the spheres 10× magnification were taken on days 4, 5, and 6, using an inverted microscope (Leica Leitz Fluovert, Wetzlar, Germany).

### 4.4. Cell Cytotoxicity Assays 

For the treatments, DOX (44583) and paclitaxel (T7402) were purchased from Sigma-Aldrich (Merck Life Science, Madrid, Spain), and liposome-encapsulated DOX (300112S) was purchased from Avanti Polar Lipids (Alabaster, AL, USA).

#### 4.4.1. MTT Cell Viability Assay

A total of 25,000 cells/well were seeded in 48-well plates and incubated overnight. Cells were then treated with the drugs (or vehicle) at the indicated times and doses. After treatment, cells were incubated with MTT reagent (M2128 Sigma-Aldrich, Merck Life Science, Madrid, Spain) at a final concentration of 0.2 mg/mL for 2 h. The mitochondrial activity of live cells was measured by spectrophotometry with a Multi-Detection Microplate Reader Synergy™HT (Bio-Rad, Hercules, CA, USA) at an absorbance wavelength of 570 nm. Percentages of viable cells were calculated, normalizing by non-treated cells in order to remove proliferation-related differences.

#### 4.4.2. Cell Viability Assay

Cell viability was evaluated using the Cell Titer-Glo® Luminescent Cell Viability Assay (G7572 Promega, Madison, WI, USA). Cells were cultured for 4 days in white 96-well plates (136101 Thermo Fisher Scientific, Waltham, MA, USA). Three-dimensional cultures were then treated with the drugs (or vehicle) at the indicated times and doses, and were processed as described by the manufacturer. Percentages of viable cells were calculated, normalizing by non-treated cells.

### 4.5. DOX Intracellular Accumulation Analysis

To measure intracellular DOX accumulation, MDA-MB-231 cells were treated with 1 µg/mL DOX. After incubation, cells were washed with phosphate-buffered saline (PBS), pelleted by centrifugation, and analysed using a FACScalibur FlowCytometer (Becton Dickinson, Rutherford, NJ, USA). 

For the pharmacokinetic analysis, cells were incubated with the drug during 4 or 8 h. The medium was then replaced by a drug-free medium and cells were further incubated until 24 h. Median intensity values were stored in 2 different datasets: drug uptake data (0–4 or 0–8 h) and drug release data (4–24 or 8–24 h). Values were normalized by initial DOX cellular content, and slopes for the datasets were calculated by linear regression. 

For the DOX imaging experiments, cells grown on poly-L-lysine-coated coverslips were treated with DOX for 2 h 30 min before fixation with 3.7% PFA for 15 min at 4 °C. Cells were then washed twice with PBS and stained with Hoechst for 5 min. After 2 further PBS washes, slides were mounted in a Fluoromount medium. Fluorescence images were acquired with a confocal laser microscopy system with a 60x oil objective (Confocal Leica TCS SP8). Argon lasers with 488 and 568 lines were used. Around 20 stacks (1 µm z-distance) were obtained for each imaged cell using identical settings. To measure DOX intensity in the nucleus compartment, the mean intensity of DOX staining in the 3D Hoechst-englobing region of interest (ROI) was quantified using the IMARIS 9.3 software (Bitplane) cell analysis tool. The same threshold value was used for all the samples.

### 4.6. Isolation of PM-Enriched Fractions 

To obtain PM-enriched fractions of MDA-MB-231 cells, the protocol described by Suski et al. [56] was optimized for cultured cells. A total of 25 million MDA-MB-231 cells in each condition were seeded in 10 plates (150 mm). After 4 days, cells were washed, scraped, and collected in PBS. Cell collection was done by centrifugation at 700× *g* for 5 min at 4 °C. Pellet was resuspended in isolation buffer (225 mM mannitol, 75 mM sucrose, 0.5% (*w*/*v* FA-free BSA (126575 Sigma, Merck Life Science, Madrid, Spain), 0.5 mM EGTA and 30 mM Tris-HCl, pH 7.4, supplemented with protease and phosphatase inhibitor cocktails (Roche, Basel, Switzerland). Homogenization was performed by pipetting 25 times up and down, using dounce homogenization (25 times with loose pestle and 25 times with tight pestle), and sonication (25 pulsations at medium power 60 A and 0.6). Sample lysate was then centrifuged at 800× *g* for 5 min at 4 °C. Supernatant was collected and pellet was resuspended and lysed as described above. Both lysates were unified and a small amount was collected as the total fraction for lipidome analysis. The rest of the sample lysate was centrifuged at 800× *g* for 5 min at 4 °C. The pellet was discarded and the supernatant was centrifuged at 10,000× *g* for 10 min at 4 °C. The pellet was again discarded and the supernatant was again centrifuged to remove any mitochondrial contamination. The clean supernatant was centrifuged at 25,000× *g* for 20 min at 4 °C to obtain the crude PM fraction. The pellet containing the crude PM fraction was gently resuspended in 500 µL of isolation buffer and centrifuged at 25,000× *g* for 20 min at 4 °C to remove microsomal and cytosolic contamination. The PM pellet was gently resuspended again in 50 µL of isolation buffer for lipidome analysis. To ensure cellular fractionation, total and PM fraction were processed by western blot analysis.

### 4.7. Liquid Chromatography-High Resolution Mass Spectrometry (LC-HRMS) Analysis

#### 4.7.1. Glycerophospholipids and Neutral Lipids

Added to the samples was a total of 750 µL of a chloroform–methanol (2:1, *v*/*v*) solution with 0.01% butylated hydroxytoluene (BTH) containing internal standards (16:0 D31_18:1 phosphocholine, 16:0 D31_18:1 phosphoethanolamine, 16:0 D31-18:1 phosphoserine, 17:0 lyso-phosphocholine, 17:1 lyso-phosphoethanolamine, 17:1 lyso-phosphoserine, 17:0 D5_17:0 diacylglycerol, 17:0/17:0/17:0 triacylglycerol, and C17:0 cholesteryl ester, 0.2 nmol each; Avanti Polar Lipids). Samples were vortexed and sonicated until they appeared dispersed and were then extracted at 48 °C and cooled overnight. Samples were then evaporated to dryness and stored at −80 °C until analysis. Before analysis, 150 µL of methanol was added to the samples, which were centrifuged at 13,000× *g* for 5 min, and 130 µL of the supernatant was transferred to a new vial ready for injection. 

#### 4.7.2. Sphingolipids 

Added to the samples was a total of 750 µL of a methanol–chloroform (2:1, *v*/*v*) solution containing internal standards (N-dodecanoylsphingosine, N-dodecanoylglucosylsphingosine, and N-dodecanoylsphingosylphosphorylcholine, 0.2 nmol each; Avanti Polar Lipids). Samples were vortexed and sonicated until they appeared dispersed, and were then extracted at 48 °C and cooled overnight. Then, 75 µL of 1 M KOH in methanol was added, and the mixture was incubated for 2 h at 37 °C. Following addition of 75 µL of 1 M acetic acid, samples were evaporated to dryness and stored at −80 °C until analysis. Before analysis, 150 µL of methanol was added to the samples, which were centrifuged at 13,000× *g* for 5 min. and 130 µL of the supernatant was transferred to a new vial ready for injection.

#### 4.7.3. LC-HRMS

LC-HRMS analysis was performed using an Acquity ultra high-performance liquid chromatography (UHPLC) system (Waters, Milford, MA, USA) connected to a time-of-flight detector (LCT Premier XE). Full scan spectra from 50 Da to 1800 Da were acquired, and individual spectra were summed to produce data points each of 0.2 s. Mass accuracy at a resolving power of 10,000 and reproducibility were maintained using an independent reference spray via the LockSpray interference. Lipid extracts were injected onto an Acquity UHPLC BEH C8 column (1.7 µm particle size, 100 mm × 2.1 mm; Waters, Milford, MA, USA) at a flow rate of 0.3 mL/min and a column temperature of 30 °C. The mobile phases were methanol with 2 mM ammonium formate and 0.2% formic acid (A)/water with 2 mM ammonium formate and 0.2% formic acid (B). A linear gradient was programmed as follows: 0.0 min: 20% B; 3 min: 10% B; 6 min: 10% B; 15 min: 1% B; 18 min: 1% B; 20 min: 20% B; 22 min: 20% B. 

Positive identification of compounds was based on accurate mass measurement (error < 5 ppm) and LC retention time compared with that of a standard (92%). 

Quantification was performed using the extracted ion chromatogram for each compound using 50 mDa windows. The linear dynamic range was determined by injecting mixtures of internal and natural standards as indicated above. Since standards for all identified lipids were not available, the amounts of lipids are given as pmol equivalents relative to each specific standard.

### 4.8. Immunoblotting

All protein samples were previously diluted with loading buffer (Tris 1M, 20% SDS, 30% glycerol and bromophenol) and heated for 5 min at 95 °C. Proteins were separated using 7.5%, 10%, or 4–12% SDS-PAGE electrophoresis and transferred to a PVDF membrane (IPVH 00010 Millipore). Membranes were immersed in a blocking solution (5% nonfat milk in 0.1% Tween 20 in 1X TBS (20 mM Tris, 137 mM NaCl and 3.9 mM HCl)) and incubated overnight with the appropriate antibody at 4 °C. Mouse anti-β-actin (1:1000, ab6276), rabbit anti-cleaved caspase 3 (1:2000, ab32042), rabbit anti-cleaved PARP1 (1:1000, ab32064), rabbit anti-VDAC1-Porin (1:1000, ab34726) and rabbit anti-calreticulin (1:1000, ab2907) were from Abcam. Human anti-CPT1C (1:500; 66072-1-Ig) was from Proteintech. Mouse anti-β-tubulin (1:2000; T5201) was from Sigma-Aldrich, (Merck Life Science, Madrid, Spain). Mouse anti-Na+K+ATPase (1:500; sc-58628) was from Santa Cruz. Following washing, the membranes were incubated with horseradish peroxidase-tagged secondary anti-mouse or anti-rabbit (1:10,000; DAKO) antibodies in TBS-T (1 h at room temperature). Blots were developed using Luminata Forte (Millipore WBLUF0500) and SYNGENE GBOX, and were quantified using ImageJ software. 

### 4.9. Quantitative Real-Time Polymerase Chain Reaction (RT-PCR) Assays

Total RNA was isolated from cultured cells using TRIzol™ Reagent (15596018 Thermo Fisher Scientific, Waltham, MA, USA) following the manufacturer’s instructions. The quantity and quality of isolated RNA was determined by Multi-Detection Microplate Reader Synergy™HT (Bio-Rad, Hercules, CA, USA).

For gene expression analysis, template cDNA was synthetized from 1 µg of total RNA using a reaction mixture composed of RT buffer (F88903, Lucigen, bioNova Cientifica, Madrid, Spain), dNTPs (D7295, Sigma, Merck Life Science, Madrid, Spain), random RT primers (309080, Exiqon, bioNova Cientifica, Madrid, Spain), M-MuLV reverse transcriptase (30222, bioNova Cientifica, Madrid, Spain), and reverse transcriptase (30281-1, bioNova Cientifica, Madrid, Spain) in a total volume of 20 µL. The mixture was incubated for 1 h at 37 °C. Quantitative PCR was performed using SYBR Green I Master Mix (1725271, Bio-Rad, Hercules, CA, USA). 

A total of 2 µL of cDNA was used to perform the PCR with the mix SsoAdvanced SYBRGreen Supermix (1725261 Bio-Rad, Hercules, CA, USA) for quantitative RT-PCR (CFX96 Real-time System; Bio-Rad, Hercules, CA, USA). The following gene-specific intron-skipping primers (IDT DNA Technologies, Leuven, Belgium) were used at 10 µM: CPT1C (for-GGA CTG ATG GAG AAG ATC AAA GA, rev-CAC AAA CAC GAG GCA AAC AG); and β-actin (for-CGT GAT GGT GGG CAT GGG TC, rev-ACG GCC AGA GGC GTA CAG GG). Relative gene expression was calculated using the comparative Ct (2^−ΔΔct^) method in relation to β-actin levels.

### 4.10. Kaplan–Meier Plotter Analysis

To assess the prognostic value of CPT1C and other functionally linked genes, we used a database that integrates gene expression and clinical data to obtain survival information for gastric, lung, breast, and ovarian cancer in relation to expression levels of genes (http://kmplot.com/analysis/ 22 February 2022) [57]. Gene expression data obtained through gene-chip data-sources from the GEO, EGA, and TCGA databases were converted into Kaplan–Meier plots. Depending on the median expression levels of CPT1C, the samples were divided into a high-expression group and a low-expression group, and the 95% confidence interval (CI), log-rank *p*-value and hazard ratio (HR) were calculated. 

### 4.11. ROC Plotter Analysis 

A ROC plotter web application (www.rocplot.org 22 February 2022) was used to link CPT1C gene expression with response to therapy using transcriptome-level data for patients with BC, filtered for patients who received anthracycline therapy. Therapy response was determined using cPR.

### 4.12. Statistical Analysis

All results are reported as mean or median ± standard error (SEM) or standard deviation (SD). Statistical tests were performed using the GraphPad Prism 6.0 software. Depending on normality (Shapiro–Wilk test and D’Agostino–Pearson test), Student’s *t* test, Mann–Whitney U test or Wilcoxon test were used to compare only 2 groups. Two-way analysis of variance (ANOVA) was performed followed by the Bonferroni post-hoc test if distinct groups including different variables were compared. Analysis of covariance (ANCOVA) was used to compare linear regression lines.

## Data Availability

Not applicable.

## Supplementary Materials

The following supporting information can be downloaded at: https://www.mdpi.com/article/10.3390/ijms24020946/s1.
