# Supplementary material for: Cpt1c Downregulation Causes Plasma Membrane Remodelling and Anthracycline Resistance in Breast Cancer
Source: Int J Mol Sci. 2023 Jan 4;24(2):946. doi: 10.3390/ijms24020946 (PMC9864098; doi:10.3390/ijms24020946)

Cpt1c downregulation causes plasma membrane remodelling and anthracycline resistance in breast cancer

Supplemental Figures

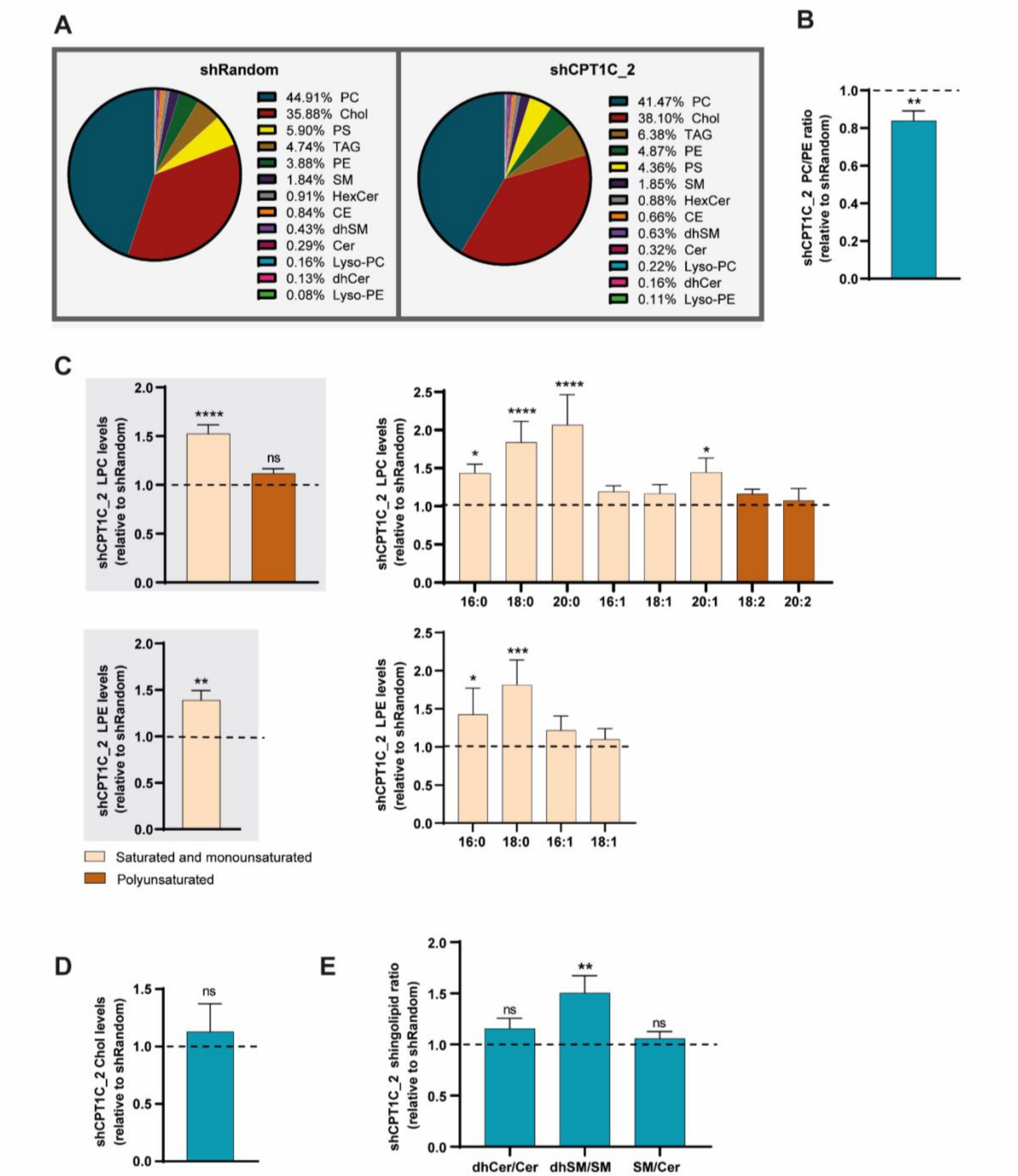

Supplemental Figure S1. Relative abundance of lipid molecular species in control and CPT1C-silenced MDA-MB-231 cells according to LC-HRMS. MDA-MB-231 cells were infected with shRandom or shCPT1C-carrying lentivirus. **A.** Average percentages of lipid species according to LC-HRMS in control and CPT1C-silenced MDA-MB-231 cells. The pie charts show the percentage distribution of the primary lipid species in PM-enriched fractions, and on the right, show the mean percentage for each detected species. **B-E.** Relative abundance of lipid species and lipid ratios in PM-enriched fractions of CPT1C-silenced MDA-MB-231 cells relative to control cells (value=1). In **C,** values are grouped by FA chain length and saturation. **B, C (left), D, E.** Results are shown as mean ± SEM (± SD in **D**) for 3 independent experiments (Wilcoxon signed-rank test; **p<0.01, ****p<0.0001). **C (right).** Results are shown as mean ± SD for 3 independent experiments (2-way ANOVA followed by Dunnett's multiple comparison test; *p<0.05, ***p<0.001 ****p<0.0001). PC: phosphatidylcholine; Chol: cholesterol; PS: phosphatidylserine; TAG:

triglycerides; PE: phosphatidylethanolamine; CE: cholesteryl esters; SM: sphingomyelin; HexCer: hexosylceramide; Cer: ceramide; dhSM: dihydrosphingomyelin; LPC: lysophosphatidylcholine; dhCer: dihydroceramide; LPE: lysophosphatidylethanolamine.

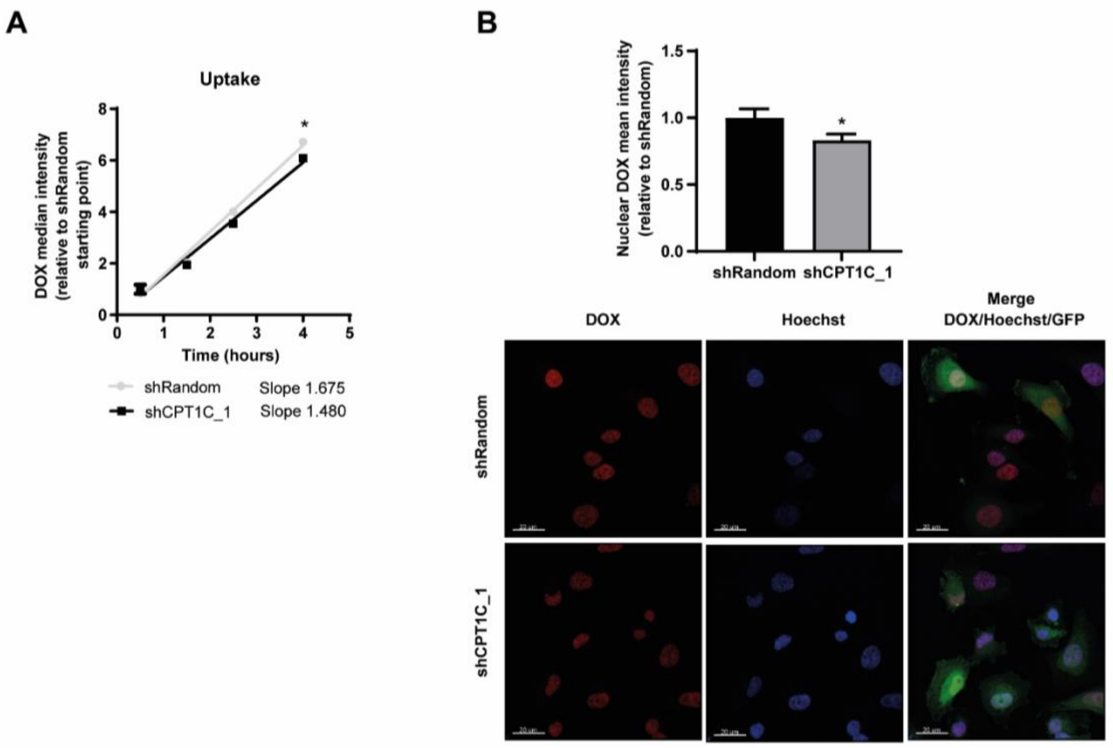

Supplemental Figure S2. CPT1C silencing decreases DOX cellular and nuclear accumulation by decreasing uptake. MDA-MB-231 cells were infected with shRandom or shCPT1C_1-carrying lentivirus. **A.** Quantification of intracellular DOX at the indicated times as detected by flow cytometry. Cells were treated with 1 μg/ml DOX for 4 h. DOX uptake results are shown as median ± SD of 2 independent experiments performed with 4 biological replicates. Slopes were compared (n=8, ANCOVA for slope comparison; uptake *p<0.05). **C (top).** Intracellular localization of DOX visualized by confocal microscopy 2 h 30 minutes after DOX exposure. Nuclei were labelled with Hoechst (scale bars, 20 μm). **C (bottom).** Quantification of fluorescence intensity (DOX) in the nuclei. Results are shown as mean ± SEM (n=34-42 cells; Student's t test; *p<0.05).

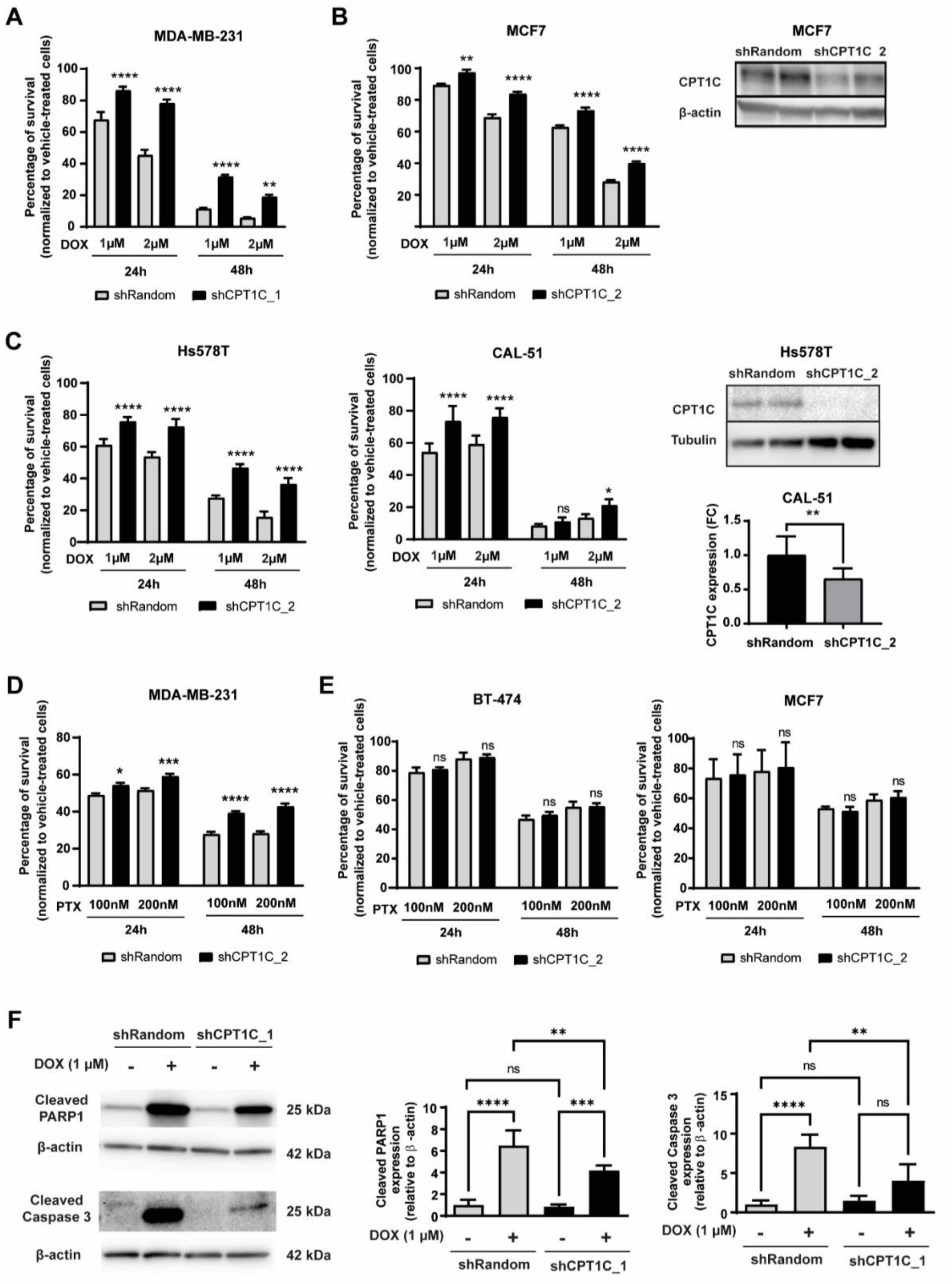

Supplemental Figure S3. CPT1C silencing increases resistance to DOX and paclitaxel in BC cells. MDA-MB-231 (**A,D**), MCF7 (**B,E**), Hs578T (**C**), CAL51 (**C**) and BT-474 (**E**) cells, were infected with shRandom, shCPT1C_1, or shCPT1C_2-carrying lentivirus. **A, B, C.** The MTT assay evaluation of BC cell line chemosensitivity to DOX after 24 or 48 hours of treatment at the indicated doses. Results are represented as mean ± SEM for 3 experiments (n=12;

2-way ANOVA followed by Bonferroni's multiple comparison test; $^{**}p<0.01$, $^{****}p<0.0001$) for (A) and mean ± SD for 2 experiments (n=8; 2-way ANOVA followed by Bonferroni's multiple comparison test; $^{*}p<0.05$, $^{**}p<0.01$, $^{****}p<0.0001$) for (B, C). CPT1C silencing was confirmed by western blot or qPCR using β-actin or β-tubulin as a loading control (B and C). **D.** The MTT assay evaluation of MDA-MB-231 chemosensitivity to paclitaxel (PTX) after 24 or 48 h of treatment at the indicated doses. Results are represented as mean ± SEM for 3 experiments (n=12; 2-way ANOVA followed by Bonferroni's multiple comparison test; $^{*}p<0.05$, $^{***}p<0.001$, $^{****}p<0.0001$). **E.** The MTT assay evaluation of BT-474 and MC7 cell chemosensitivity to PTX after 24 or 48 h of treatment at the indicated doses. Results are represented as mean ± SD for 4 and 2 experiments, respectively (n=16 and n=8, respectively; 2-way ANOVA followed by Bonferroni's multiple comparison test). **F.** Western blot analysis of apoptosis-associated proteins (cleaved PARP1 and cleaved caspase 3). Cell lysates were prepared after 24 h of DOX treatment. CPT1C silencing was confirmed and β-actin was used as a loading control. Representative results are shown as mean ± SD (n=4 per condition; 1-way ANOVA followed by Dunn's multiple comparison test; $^{**}p<0.01$, $^{***}p<0.001$, $^{****}p<0.0001$).

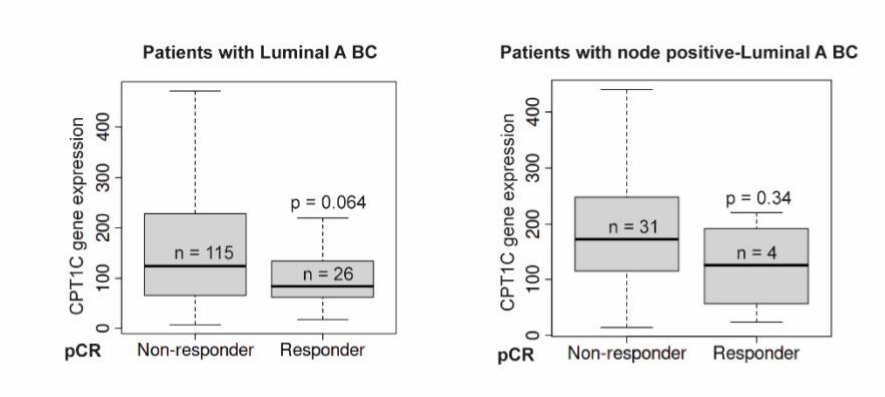

Supplemental Figure S4. CPT1C expression and survival in patients with Luminal A BC. ROC plotter analysis to evaluate CPT1C expression versus therapy response (based on pCR) using transcriptome-level data of patients with BC, filtered for anthracycline-treatment for luminal A (left) and node-positive luminal A (right). Box-and-whisker plots represent the median, minimum, and maximum values of CPT1C expression for responding and nonresponding patients according to pCR.

Original uncropped Western blots of Figure 1, Figure 3, and Supplemental Figure S3

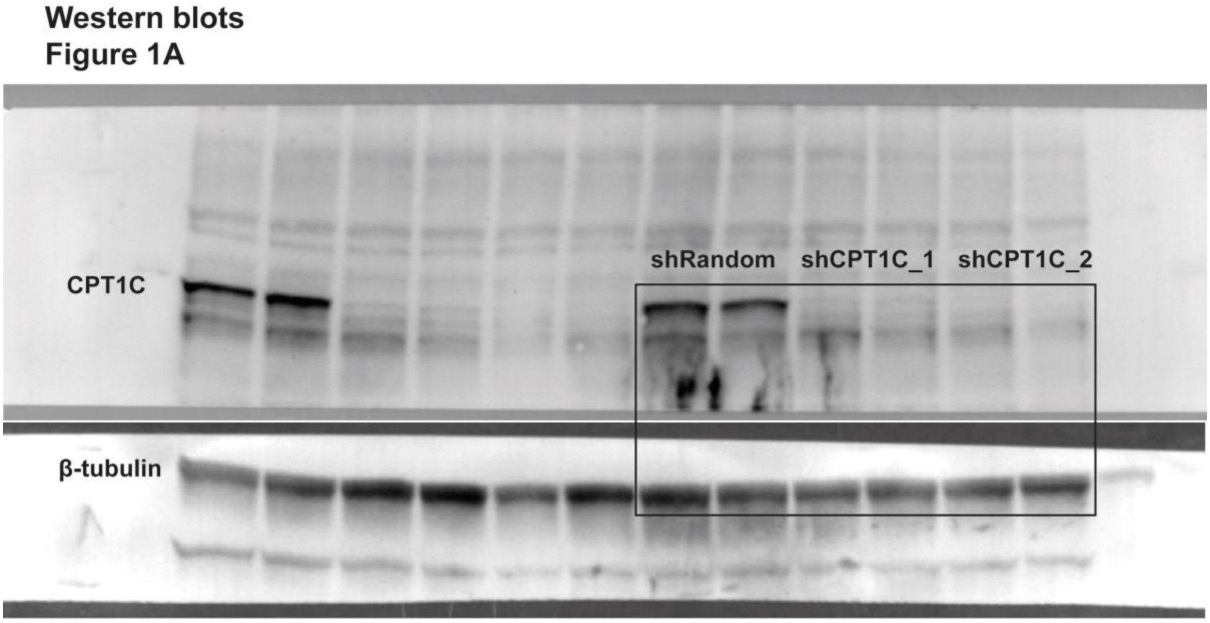

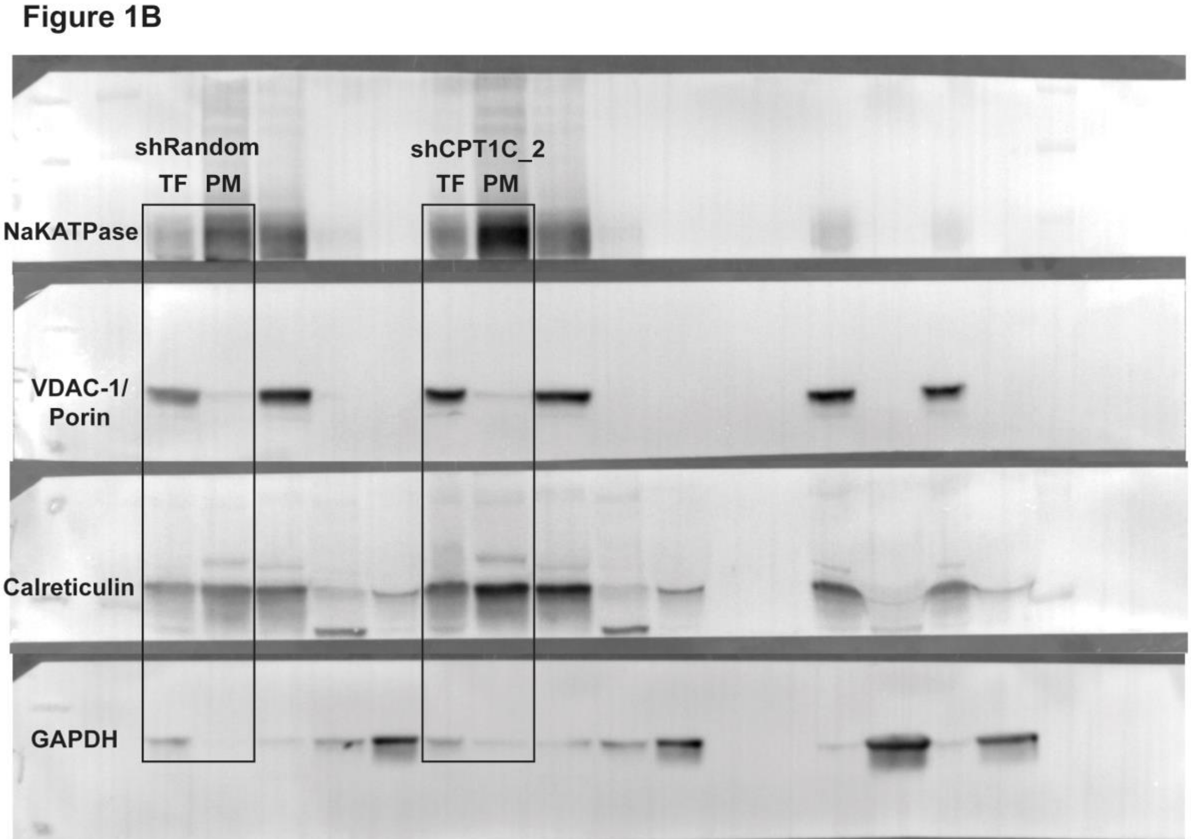

Figure 3A

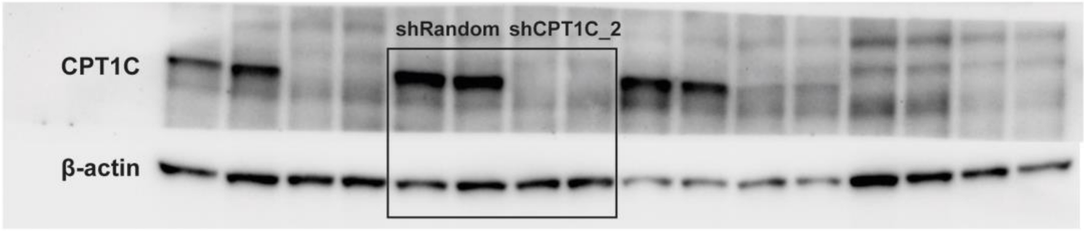

Figure 3B

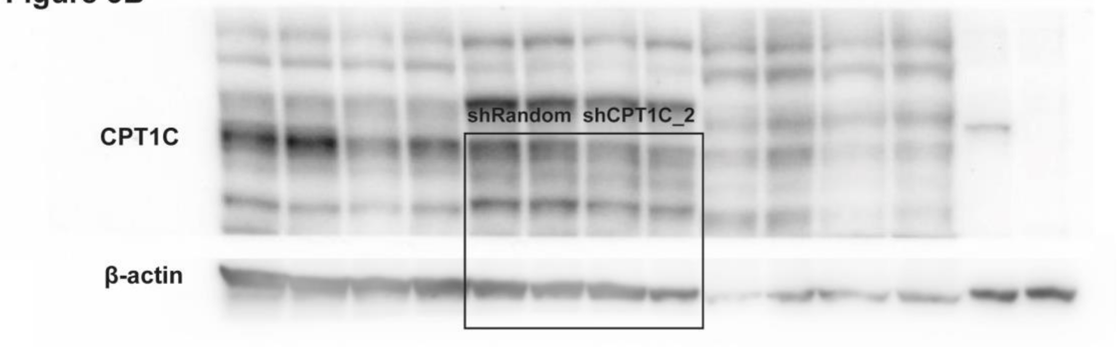

Figure 3E

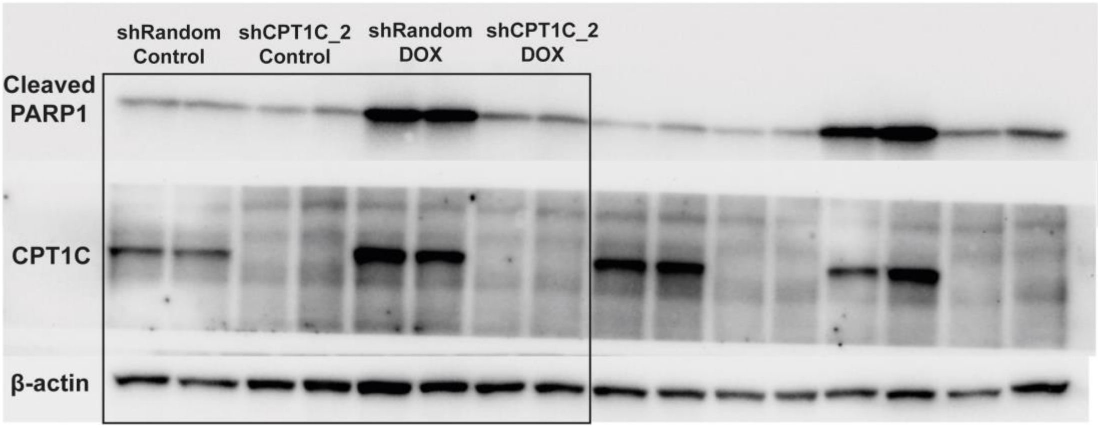

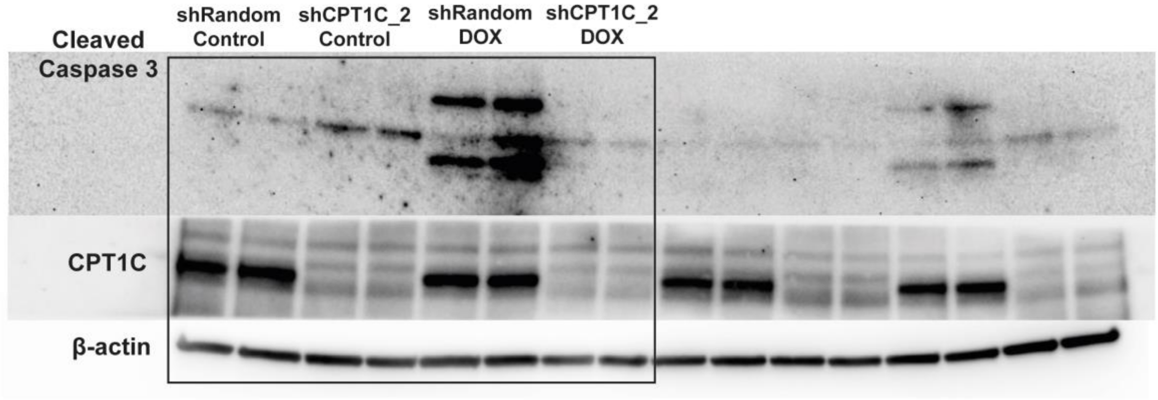

Supplemental data 3B

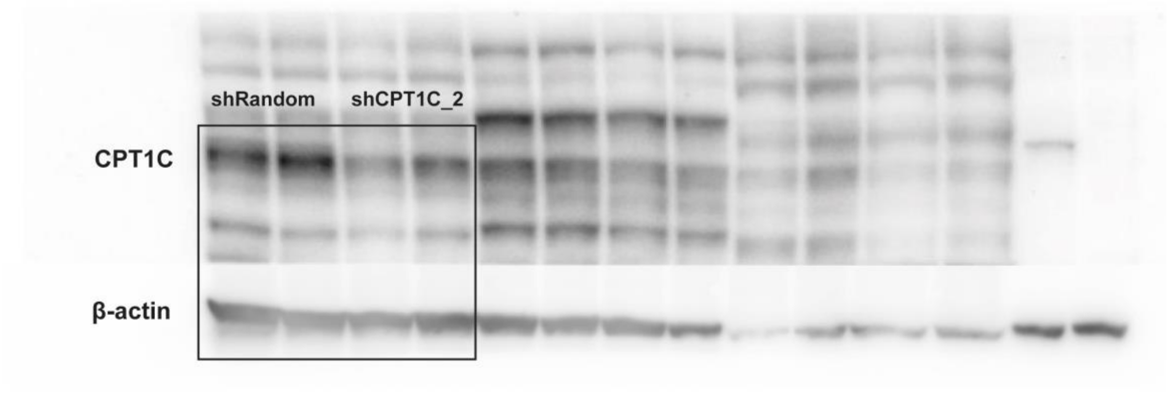

Supplemental data 3C

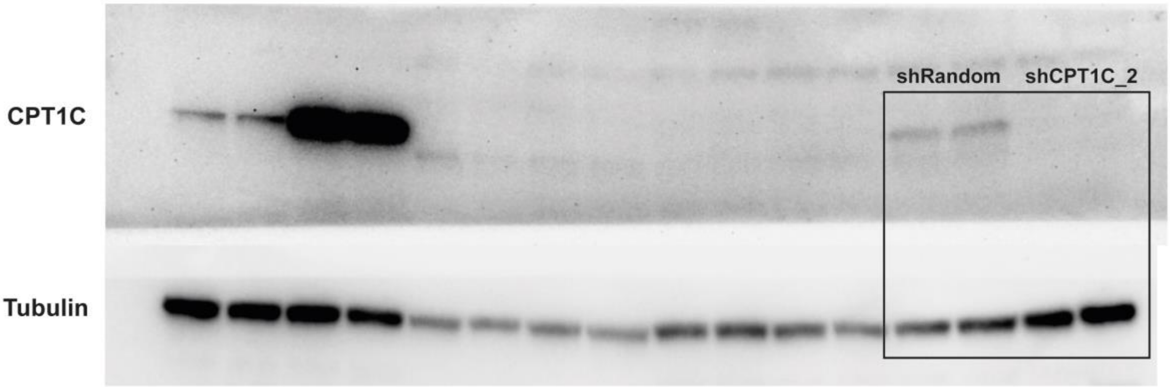

Supplemental data 3F

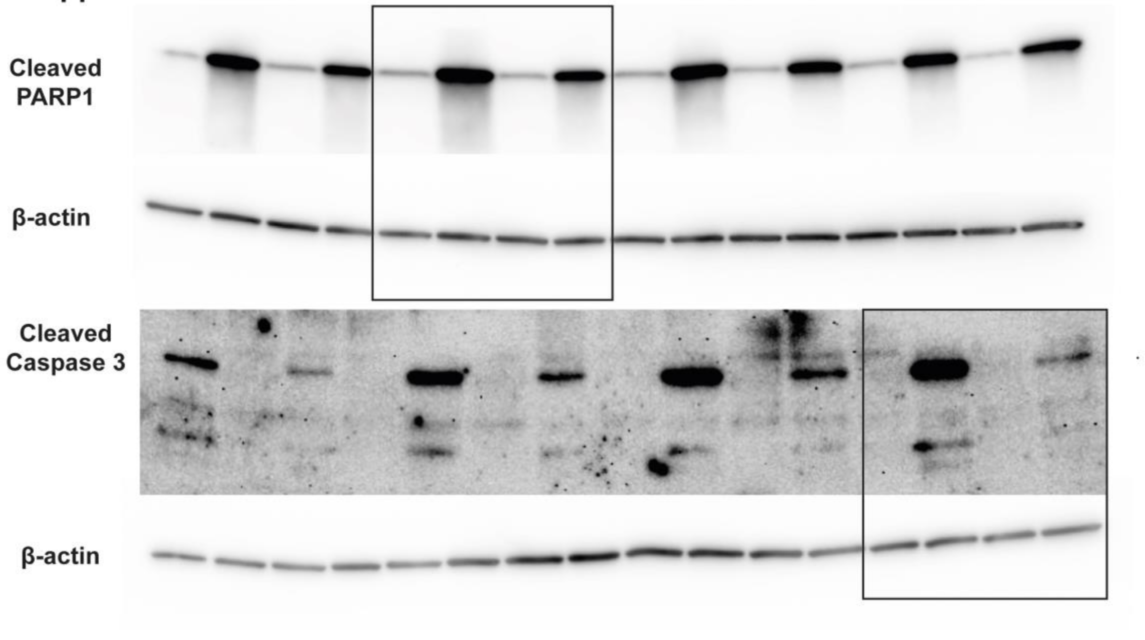

Supplement: Supplementary file 1 [file ijms-24-00946-s001.zip › ijms-2062761-supplementary.pdf]
